# Role of a Structured Physical Activity Pathway in Improving Functional Disability, Pain and Quality of Life in a Case of Breast and Gynecological Cancer Survivorship

**DOI:** 10.3390/jcm8040531

**Published:** 2019-04-18

**Authors:** Daniela Mirandola, Maria Grazia Muraca, Eleonora Sgambati, Mirko Manetti, Mirca Marini

**Affiliations:** 1 Department of Experimental and Clinical Medicine, Section of Anatomy and Histology, University of Florence, 50134 Florence, Italy; daniela.mirandola@unifi.it (D.M.); mirko.manetti@unifi.it (M.M.); 2 Oncological Network, Prevention and Research Institute (ISPRO), 50139 Florence, Italy; m.muraca@ispo.toscana.it; 3 Department of Biosciences and Territory, University of Molise, 86090 Pesche, Italy; eleonora.sgambati@unimol.it

**Keywords:** physical activity, cancer pain, functional disability, quality of life, cancer survivor

## Abstract

Physical activity (PA) interventions can improve physical functioning, treatment-related symptoms and quality of life (QoL) in cancer survivors. Most investigations have been conducted in breast cancer survivors, while studies on PA interventions in gynecological cancer survivors are scant. Here, we report for the first time the possible benefits of a structured PA pathway (i.e., eight weeks of adapted PA followed by twelve weeks of adapted fitness) on physical side effects, pain and QoL in an uncommon case of survivorship of both primary breast and gynecological cancers. For this purpose, a 69-year-old woman was assessed by functional test battery (shoulder–arm mobility, range of motion, back flexibility) at baseline and after the structured PA pathway. QoL and surgical shoulder, back and lower limb pain intensity were evaluated by Short Form-12 (SF-12) and numerical rating scale questionnaires, respectively. Lower limb circumference was also assessed. Improvement in upper limb function, reduction of lower limb edema and pain perception, as well as an increase in overall QoL were achieved after the completion of structured PA intervention. Our findings suggest that a PA intervention tailored to individual characteristics may represent an effective countermeasure to reduce post-treatment functional disability and pain, and thus to improve QoL in breast and gynecologic cancer survivors.

## 1. Introduction

Current cancer treatment appears to be increasingly effective in improving patient survival, though it can produce short- and/or long-term negative physical and psychological effects which heavily compromise the patient’s quality of life (QoL) [1,2].

In particular, breast cancer is currently the neoplasm with the highest incidence among women worldwide [3]. Breast cancer treatment complications can include decreased shoulder range of motion (ROM), lymphedema, muscle weakness, postural changes, mood disturbance, anxiety, and depression, overall negatively affecting QoL [1,2]. In breast cancer survivors, pain may persist with an uncertain prognosis in the long term, causing difficulty in everyday task performance or even severe disability [1,2].

Furthermore, endometrial cancer is the fourth most common female cancer [3] and, among the gynecological cancers, it is the most prevalent pelvic malignancy [4]. Regarding treatment-related outcomes that can affect women cured for endometrial cancer, lower limb lymphedema (LLL) is a chronic, progressive and incurable condition often leading to disability [5,6,7]. Specifically, secondary LLL is the result of lymphatic system insufficiency and impaired lymph transport with increased limb volume related to cancer treatments such as surgical removal of the pelvic and para-aortic lymph nodes (i.e., lymphadenectomy) and radiation [8,9]. The risk of developing LLL can be any time between the immediate surgery and many years thereafter [10]. It can be asymptomatic or associated with symptoms including swelling, erythema, lumps, tightness, pain, and heaviness in the leg with possible impairment in the ability to walk. This complication requires lifelong treatment and psychosocial support due to adverse effects on physical, psychological, esthetic and emotional wellbeing [10,11]. In addition, lower extremity physical dysfunction and pain negatively influence the woman’s ability to care for her family and work, negatively affect body image, limit social life, cause inability to perform everyday activities or severe disability, thus strongly jeopardizing QoL of cancer survivors [6,9,12,13]. Moreover, these impairments may negatively affect healthy lifestyle behaviors such as regular exercise [14]. Therefore, LLL requires lifelong management either to improve the condition or prevent its worsening [11].

Although recent research supports the positive effects of physical activity (PA) programs for cancer survivors with a significant improvement of QoL [1,2,14,15,16], most studies on secondary lymphedema have been performed on the upper limb after breast cancer [17,18]. Despite the clinical findings, studies exploring the role of exercise interventions for LLL consequent to gynecological cancer treatment are scant [18]. In particular, a pilot study reported the feasibility of weightlifting in a small group of patients with LLL secondary to cancer, albeit an increase in muscle strength and limb mobility was not accompanied by a reduction of LLL and a better QoL [18]. Of note, the anatomo-functional and clinical differences between upper and lower limb lymphedema after cancer treatment preclude the assumption that the success of exercise interventions for breast cancer survivors can be translated to those at risk for LLL. For this reason, it is of fundamental importance a careful case evaluation and the design of a structured PA intervention that could be tailored to the specific subject’s needs is made. Furthermore, to the best of our knowledge, previous reports have not described adapted PA (APA) programs in both breast and gynecological cancer survivors. In this context, the purpose of our study was to evaluate the possible effects of a structured PA pathway in improving cancer- and treatment-related sequelae (i.e., reduction of LLL and improvement of pain and QoL) in a case of survivorship of both primary breast and gynecological cancers. Given that we have previously demonstrated the effectiveness of a specific APA protocol in breast cancer survivors [1], our intent was also to provide groundwork for designing future research aimed at developing effective PA interventions for women with negative outcomes from gynecological cancer treatments.

## 2. Case Report

### 2.1. Case Presentation

A 69-year-old married woman with a previous clinical history of both primary breast and endometrial cancers was referred to the Cancer Rehabilitation Center (Ce.Ri.On) in Florence for cancer-related follow-up management. The survivor was included in the Ce.Ri.On waiting list for possible rehabilitation programs, and then randomly recruited to initiate APA intervention. Tracing the clinical history, in May 2008 she was diagnosed with breast cancer (i.e., ductal carcinoma in situ) and underwent bilateral mammoplasty after right lumpectomy with radiation therapy. Moreover, in August 2015 after a diagnosis of invasive endometrial adenocarcinoma, the patient underwent total extrafascial hysterectomy by laparoscopic approach, bilateral ovariosalpingectomy and bilateral pelvic lymphadenectomy removing ten and eight lymph nodes at left and right side, respectively. Subsequently, the patient was treated with combinations of chemotherapy and either internal or external pelvic radiotherapy. Following radiotherapy, LLL developed. About two years after hysterectomy, the patient underwent further surgery for local recurrent disease with removal of the pelvic back wall without adjuvant treatment. In December 2017, on the basis that there was no medical contraindication, the Ce.Ri.On rehabilitation physician recommended the woman’s participation in a well-planned and structured PA pathway (i.e., eight weeks of APA followed by twelve weeks of adapted fitness (AF)) to attenuate and improve cancer- and treatment-related problems with particular attention to LLL. Lymph drainage was also scheduled to treat LLL, but it was not performed because of patient’s personal reason at the call time. Therefore, the woman participated in a structured PA intervention without prior physiotherapy management.

Physical examination detected more pronounced left LLL and fibrosis than at the contralateral lower limb. Physical assessment and the eight-week APA program were carried out at the Ce.Ri.On center between January 2018 and March 2018. After ending APA, an AF protocol was properly continued outside the Ce.Ri.On from April to June 2018. At baseline, during and after ending the structured PA intervention, the woman underwent a functional test battery to assess the mobility of upper limb joints (active ROM test and muscle length test) and the flexibility of the spine (sit-and-reach test) [1,2,19]. In particular, active ROM was evaluated by goniometry with the subject standing and taking into account the extension (normal reference range 0–45), flexion (normal reference range 0–180), abduction (normal reference range 0–180), and external rotation (normal reference range 0–90). Shoulder mobility was assessed with the muscle length test executed with the subject in a supine position by elevating the arm and measuring the distance (cm) from the lateral epicondyle to the surface (i.e., a smaller distance from the surface corresponding to a better mobility function). The sit-and-reach test is an indirect measure of both hamstring musculotendinous unit length (indirect measure of hip ROM) and of lumbar ROM. To perform the test, the subject sat with extended legs as straight as possible, then flexed their hip joints and vertebral column to reach forward as far as possible and touched their toes in dorsiflexion with the fingertips of both hands simultaneously. A meter rule was placed between the legs with 0 cm located at the heel line to measure the distance from toes to hands. Moreover, the subject filled out the Short Form-12 (SF-12) and the numerical rating scale (NRS) questionnaires to assess the QoL and to quantify shoulder, lower limb and back pain intensity, respectively. Finally, both lower limbs were assessed by multiple circumference measurements at several anatomical points from foot to hip with a thin, flexible tape [20]. A difference between affected and unaffected lower limb girth of ≥3 cm at any measurement point was considered indicative of moderate/severe LLL. Anthropometric parameters, such as height and weight, were also measured at baseline and after ending the structured PA intervention and used to calculate body mass index (BMI, kg/m^2^). The study was carried out following the rules of the Declaration of Helsinki of 1975 (https://www.wma.net/what-we-do/medical-ethics/declaration-of-helsinki/), revised in 2013. Following that, no ethics committee was needed for this publication and the Italian Protection of Personal Data Law (D. Lgs. n. 196/2003) was complied. A signed informed consent form was obtained from the participant.

### 2.2. Structured Physical Activity Pathway

The structured PA pathway, planned by an adapted exercise specialist on the basis of the specific patient’s needs, comprised eight weeks of APA followed by twelve weeks of AF. In particular, a reduction of LLL, an increase in the shoulder mobility, and an overall improvement of pain and QoL were considered specific objectives to achieve through the structured PA pathway. The APA protocol consisted of one-hour sessions performed during two nonconsecutive days per week and was organized into three phases as previously described [1]. In the first phase (from the 1st to the 5th session), breathing exercises and exercises to mobilize the pelvis and stretching were proposed, including specific exercises to preserve or improve upper limb mobility. In the second phase (from the 6th to the 12th session), the upper limb mobility exercises of the first phase were replicated, increasing the working time. Postural exercises were also proposed according to the active posture reeducation [1]. The third phase (from the 13th to the 16th session) comprised a circuit training workout including alternated low-intensity and higher workload exercises. Postural exercises were also replicated. Because of the patient’s lower limb impairment, specific exercises to increase ankle and hip active ROM and, therefore, make the subject able to start executing squats and lunges were also included in the second and third phases. All sessions started with a 10-min warm-up of general exercises and ended with a 10-min cool-down of stretching and breathing exercises. After ending the eight-week APA, the woman voluntarily participated in an AF program which is proposed by Ce.Ri.On as enhanced PA to increase physical fitness and then possibly start a sport activity. In particular, the twelve-week AF program was performed in one-hour sessions during two nonconsecutive days per week. PA was divided into three phases and each phase consisted of warm-up, adapted training and cool-down as detailed in Table 1. Each exercise was carefully described through simple biomechanical concepts and visually shown by the exercise specialist. The warm-up and the cool-down phases were the same for all fitness program phases. The warm-up consisted of 20 min of general aerobic exercises for major muscle groups while the cool-down included 15 min of stretching and breathing exercises. The central adapted training lasted about 25 min and was organized as follows. In the first phase (from the 1st to 3rd week), the AF program was aimed to provide continuity to the activities previously executed during APA. First, specific walking pattern exercises were proposed. Ankle and hip mobility exercises were to be performed either in a standing position or on the floor. In addition, exercises such as squats and lunges were continued. Beginner core exercises were also proposed. In the second phase (from 4th to 8th week), lower limb resistance and strength training were included (i.e., exercises for hip and leg flexor, extensor, abductor, and adductor muscles). Throughout the four weeks a progressive resistance exercise program was applied, and thus all bodyweight exercises were performed in 3 sets of 5–12 repetitions. Beginner core exercises were replicated. In the last phase (from the 9th to 12th week), coordination and balance exercises were proposed in circuit training to increase the difficulty of each exercise. Moreover, exercises included the use of resistance bands and a small gym ball, in order to improve the fitness performance. Core exercises were implemented. Each exercise was carefully described through basic biomechanical concepts and visually shown by the exercise specialist. The patient engaged well with all the proposed sessions and, therefore, the adherence to the PA pathway was complete (i.e., the patient attended 100% of scheduled PA sessions).

## 3. Results

Table 2 shows data concerning the functional test battery evaluation and self-reported questionnaires (NRS and SF-12) of case study at baseline and after the structured PA pathway. The results obtained either after an eight-week APA or a twelve-week AF program, both constituting the PA intervention, are displayed (Table 2). Specifically, the ROM values of both surgical and non-surgical upper limbs at baseline fell within the normal reference range and were preserved after the structured PA intervention (Table 2). Conversely, the muscle length test revealed that mobility of either the surgical or non-surgical shoulder at baseline was compromised, with an evident asymmetry consisting in a 10-cm worse value for non-surgical shoulder. After the structured PA intervention, a progressive bilateral gain in shoulder mobility accompanied by an evident reduction in the difference between the surgical and non-surgical shoulder respect to baseline was observed (Table 2). Furthermore, low back flexibility, assessed by the sit-and-reach test, showed an evident improvement following the PA intervention (Table 2). We also observed a trend toward an improvement in back pain after PA intervention as well as a disappearance of surgical shoulder and lower limb pain. Finally, the higher than at baseline SF-12 scores for either the physical or mental components achieved post-PA intervention highlighted a trend toward a better QoL (Table 2).

The results of lower limb circumference measurements using anatomical landmarks are reported in Table 3. At baseline, the patient presented with mild LLL. Although the differences between the right and left lower limb girth increased after structured PA intervention, a relevant decrease in circumference of both lower limbs was detected (Table 3).

Finally, no musculoskeletal injuries resulted from the structured PA intervention and no difference in BMI was detected between baseline and after ending the structured PA pathway (20.2 kg/m^2^ and 20.3 kg/m^2^, respectively).

## 4. Discussion

In the current study, we present a structured, safe, and effective PA intervention to manage chronic complications resulting from treatments in an uncommon case of survivorship to both primary breast and gynecological cancers. Advances in treatment techniques have led to improved survival rates in patients with cancer. Consequently, patients are living longer with possible physical and psychological impairments that result from their disease or its treatments [1,2,5]. To the best of our knowledge, this is the first case report describing the benefits of a specific PA intervention, consisting of an eight-week APA followed by a twelve-week AF program, on physical side effects, pain and QoL in a woman after both breast and gynecological cancer treatments. First, our results confirm that a structured exercise intervention may preserve the upper limb ROM and improve shoulder mobility. Although our patient presented at baseline with a normal upper limb ROM, likely because she underwent lumpectomy and not more invasive surgery (e.g., quadrantectomy or full-breast mastectomy), she had impaired mobility of both the surgical and non-surgical shoulders. In particular, the shoulder mobility at the non-surgical site was even worse, which might be explained by a musculoarticular overload over time due to a tendency to preserve the surgical limb. Therefore, it is noteworthy that our PA protocol was able not only to ameliorate either surgical or non-surgical shoulder mobility, but also to reduce the marked inter-limb mobility asymmetry present at baseline. Second, we herein show that the proposed PA may reduce lower limb edema and ameliorate overall QoL. Indeed, it is worth noting that the patient’s SF-12 physical and mental scores increased after the PA pathway, reaching levels comparable to those of the general healthy Italian population [21]. In addition, it is remarkable that our structured PA intervention was effective in reducing back pain intensity and eliminating surgical shoulder and lower limb painful symptoms. Increasing evidence indicates the positive effects of structured and regular PA on psychophysical outcomes of cancer survivors, especially in breast cancer survivors, supporting that PA should be part of standard care in survivorship [1,2,14,15,16]. According to literature data, in our previous investigations, we reported the efficacy of personalized programs of APA in reducing shoulder–arm complications or upper limb lymphedema, thus helping in recovering QoL [1,2,19]. As far as the secondary upper limb lymphedema is concerned, the role of PA has recently been reported in breast cancer survivors [17,18,22]. However, research examining the PA role in subjects with LLL secondary to cancer treatment is scant [18]. Only a few studies reported the occurrence of adverse effects due to the practice of physical exercise, though a possible benefit to the affected lower limb is not yet well established [18,23]. In this context, a study has evaluated the effect of an eight-week supervised progressive weight training, followed by three months unsupervised, on leg swelling and functional mobility as well as QoL in subjects with secondary LLL [18]. In particular, exercises were performed twice weekly using variable resistance machines (e.g., leg press, leg extension, leg curl, hip flexion, leg abduction, prone straight leg lifts), free weights, and ankle weights. After the exercise protocol, increased leg strength and enhanced functional mobility without improvement in QoL were reported. Of note, the LLL was unchanged [18]. At variance with the aforementioned report, here we described the effectiveness of a planned PA intervention, supervised by an exercise specialist, on the physical and psychological problems of a cancer survivor. In particular, the proposed PA intervention consisted of an eight-week APA including mainly proprioception and neuromuscular control exercises in order to improve the postural reeducation and control the pain, thus optimizing the overall QoL.

Each exercise was carefully described through biomechanical simple concepts and visually shown by the exercise specialist, and subsequently the woman performed the exercises under the specialist supervision. Additional information by direct visual and tactile feedback was used to improve the subject’s perception of their personal body image. In order to increase the visual perception of the body during exercise, mirrors were also used. The APA was followed by a twelve-week AF program characterized by muscle strengthening exercises either to enhance physical fitness and well-being or to promote healthy lifestyle behaviors such as the participation in regular PA. It is noteworthy that our PA intervention was structured taking into account the simplicity, feasibility, effectiveness, and possible reproducibility of exercise performance in different contexts, such as in other rehabilitation centers or self-practice at home. Though our data are limited by research through a case study, we have shown an improvement in lower limb swelling with ameliorated QoL after the overall PA pathway. Instead, the interlimb circumference differences increased after the structured PA intervention. Of note, the diagnosis of LLL is challenging as signs and symptoms are often unrecognized, and it can be bilateral, thus preventing the comparison with an unaffected lower limb [11]. In fact, we reported a bilateral circumference reduction with a decrease in lower limb pain severity.

It should also be pointed out that the overall improvements obtained through our structured PA intervention have been achieved without the simultaneous use of instrumental and manual therapies, and without the influence of practicing other exercise activities outside of the PA pathway. Therefore, the positive effects herein reported may be ascribed to the effectiveness of the structured PA intervention planned, proposed and supervised by an exercise specialist. As the life expectancy for cancer survivors improves, the risk of complications resulting from treatment is increasing. Consequently, effective prevention and management of the adverse effects of surgery and therapy are receiving growing importance [2,16,22]. Hence, qualified physical exercise specialists should be included in the team of specialized expertise delivering an integrated approach to the care of patients with cancer. Due to the psychophysical benefits obtained in a short time frame, it seems of major importance for survivors to continue an appropriate exercise program [2].

## 5. Conclusions

Collectively, our findings demonstrated that upper and lower limb morbidity in a case of survivorship to both breast and gynecological cancers can be improved over time with a structured and planned PA pathway, tailored to the individual patient’s needs. These encouraging findings suggest that multidisciplinary cancer care is required to achieve the best functional, physical, and psychological outcomes. Further studies, including higher numbers of cases, a long-term follow-up and a case-control design, as well as more appropriate diagnostic assessment tools (e.g., bioelectrical impedance analysis and ultrasound techniques) to evaluate the body composition, will be necessary to develop and validate effective PA recommendations for survivors to breast and gynecological cancers.

## Figures and Tables

**Table 1:** Description of the twelve-week adapted fitness (AF) protocol.

	First Phase	Second Phase	Third Phase
	1st–3rd Week (1st–6th session)	4th–8th Week (7th–16th Session)	9th–12th Week (18th–24th Session)
**Warm-up** (20 min)	general aerobic exercises	general aerobic exercises	general aerobic exercises
**Adapted training** (25 min)	walking pattern exercises	progressive lower limb resistance	coordination and balance exercise circuit training
	ankle and hip mobilization	progressive lower limb strength training	workout with resistance band and a small gym ball
	squat and lunge exercises		
	beginner core exercises	beginner core exercises	core exercises
**Cool-down** (15 min)	stretching	stretching	stretching
	breathing exercises	breathing exercises	breathing exercises

**Table 2:** Fitness tests, pain intensity and quality of life scores at baseline and after the completion of either adapted physical activity (APA) or adapted fitness (AF) protocols.

Variables	Baseline	Post-APA	Post-AF
**ROM surgical limb**, grade			
Extension	45	45	45
Flexion	180	180	180
External rotation	90	90	90
Abduction	180	180	180
**ROM non-surgical limb**, grade			
Extension	45	45	45
Flexion	180	180	180
External rotation	90	90	90
Abduction	180	180	180
**Surgical shoulder mobility**, cm	10	3	2
**Non-surgical shoulder mobility**, cm	20	14	5
**Sit-and-reach**, cm	8	0	0
**Perception of pain (NRS)**			
Surgical shoulder pain	2	0	0
Non-surgical shoulder pain	0	0	0
Cervical pain	7	5	2
Dorsal pain	6	5	1
Lumbar pain	7	5	2
Right lower limb	2	0	0
Left lower limb	4	2	0
**Quality of life (SF-12)**			
Physical	38.4	50.7	49.8
Mental	32.6	47.2	43.1

ROM, range of motion; NRS, numerical rating scale; SF-12, Short Form-12.

**Table 3:** Measurements of lower limb circumference at several anatomical points at baseline and after adapted fitness (AF).

Lower Limb Circumference, cm	Baseline			Post-AF		
	Right	Left	Difference	Right	Left	Difference
Groin level	60	61	1	53	54.5	1.5
Midline of thigh	43	45	2	41.5	43.5	2
Midline of knee	36	37	1	35.5	37	1.5
Half leg	32	32.5	0.5	30.5	32	1.5
Ankle	22	23	1	21.5	23	1.5
Midline of foot	21	21.5	0.5	21	22	0.5
